# Genome-wide analyses of the bHLH superfamily in crustaceans: reappraisal of higher-order groupings and evidence for lineage-specific duplications

**DOI:** 10.1098/rsos.172433

**Published:** 2018-03-14

**Authors:** Wai Hoong Chang, Alvina G. Lai

**Affiliations:** Nuffield Department of Medicine, University of Oxford, Old Road Campus, Roosevelt Drive, Headington Oxford, OX3 7FZ, UK

**Keywords:** crustacean, *Litopenaeus vannamei*, bHLH, helix-loop-helix, bHLH-PAS, comparative genomics

## Abstract

The basic helix-loop-helix (bHLH) proteins represent a key group of transcription factors implicated in numerous eukaryotic developmental and signal transduction processes. Characterization of bHLHs from model species such as humans, fruit flies, nematodes and plants have yielded important information on their functions and evolutionary origin. However, relatively little is known about bHLHs in non-model organisms despite the availability of a vast number of high-throughput sequencing datasets, enabling previously intractable genome-wide and cross-species analyses to be now performed. We extensively searched for bHLHs in 126 crustacean species represented across major Crustacea taxa and identified 3777 putative bHLH orthologues. We have also included seven whole-genome datasets representative of major arthropod lineages to obtain a more accurate prediction of the full bHLH gene complement. With focus on important food crop species from Decapoda, we further defined higher-order groupings and have successfully recapitulated previous observations in other animals. Importantly, we also observed evidence for lineage-specific bHLH expansions in two basal crustaceans (branchiopod and copepod), suggesting a mode of evolution through gene duplication as an adaptation to changing environments. In-depth analysis on bHLH-PAS members confirms the phenomenon coined as ‘modular evolution’ (independently evolved domains) typically seen in multidomain proteins. With the amphipod *Parhyale hawaiensis* as the exception, our analyses have focused on crustacean transcriptome datasets. Hence, there is a clear requirement for future analyses on whole-genome sequences to overcome potential limitations associated with transcriptome mining. Nonetheless, the present work will serve as a key resource for future mechanistic and biochemical studies on bHLHs in economically important crustacean food crop species.

## 1. Background

The basic helix-loop-helix (bHLH) transcription factor superfamily is among one of the most ancient gene families shared by eukaryotic organisms [1–3]. Members of this superfamily possess two conserved yet functionally distinct domains made up of a basic DNA binding domain (E box) at the amino-terminal followed by an HLH domain at the carboxy-terminal where the latter confers the possibility for hetero- or homodimerization with other proteins [4,5]. Both domains are required to form active DNA binding complexes whereby the functionality and precision in regulating gene expression networks are determined by the formation of multiple dimer combinations through the HLH domains [6]. This dimerization potential is, arguably, an effective mechanism for gene regulation considering that each dimer pair probably has specific genetic targets. For instance, bHLHs in animals are intimately linked to the regulation of a multitude of physiological and developmental processes. Some of these include cell cycle regulation, neurogenesis, haematopoiesis, myogenesis, differentiation, apoptosis, juvenile hormone signalling in arthropods and sensing of extracellular stimuli [1,2,7–15].

Given their importance as critical transcriptional regulators in major biological processes, it is perhaps not surprising that many studies have focused on the identification and characterization of bHLH orthologues across various plant and animal species. From the first bHLH motif identified, the murine E12 and E47 transcription factors [16], full genetic complements of bHLH proteins have since been reported in various model organisms owing to the availability of complete genome sequences [3,17–25]. Phylogenetic analyses on bHLH orthologues from model bilaterian species (*Homo sapiens, Drosophila melanogaster* and *Caenorhabditis elegans*) and early branching metazoans (the demosponge *Amphimedon queenslandica* and cnidarians *Nematostella vectensi*s and *Hydra magnipapillata*) have convincingly demonstrated that diversification of metazoan bHLHs could have arisen in two steps: the first occurring before the divergence of demosponges from other animals and the second before the divergence of cnidarians and bilaterians [3,21]. Moreover, the increasing repertoire of bHLH proteins have enabled the classification of bHLH orthologues into six higher-order groups (A, B, C, D, E and F) based on distinct structural features [1,2,19,26,27].

Major efforts have thus far focused on the classification of bHLH proteins in model organisms. Beyond canonical model species, some headway has been made in elucidating bHLH genes in major metazoan lineages, which include bilaterians (deuterostomes and protostomes) and basal metazoans (hydra and sponge) [3]. Yet, relatively little is known about bHLHs in one of the most important groups of animals that represent a significant portion of aquatic sources of proteins, i.e. crustaceans. To date, studies in crustaceans are not only limited to just a few species but also to specific bHLH groups [13,14]. Systematic and cross-species characterization of crustacean bHLH proteins focusing on major food crop species from the order Decapoda is therefore necessary. In fact, numerous studies have begun to shed light on the importance of group C members, collectively known as bHLH-PAS, as environmental sensors [28]. For example, members of the crustacean bHLH-PAS family have been shown to regulate growth and reproduction through juvenile hormone signalling (Methoprene-tolerant, MET) [15,29], locomotion through circadian timekeeping (aryl hydrocarbon receptor nuclear translocator-like protein 1, BMAL1) [30,31] and response to changes in oxygen tension (hypoxia inducible factor 1, HIF-1) [32,33]. This holds much relevance for shrimp farming industries that are constantly facing the pressures of fluctuating environmental conditions that could potentially compromise aquaculture yield [32,34,35].

Here, we perform a cross-species characterization of the crustacean bHLH superfamily to address this major deficit in the field. The large number of recent crustacean transcriptomic datasets in public repositories has now permitted in-depth studies on key food crop species from the order Decapoda and other species across the broader Crustacea, hence offering important insights into the diversity of crustacean bHLHs (electronic supplementary material, table S1). Using sequence, motif and domain similarity-based approaches, we have conservatively identified 4113 bHLH orthologues, which include 3777 orthologues from 126 crustacean species and 336 orthologues from six other non-crustacean arthropod species (electronic supplementary material, tables S1 and S2 and file S1). Within this key dataset, we define higher-order bHLH groups in three major decapod species using phylogenetic-based approaches and annotate bHLH-PAS proteins in decapods and basal crustaceans (branchiopods and copepods).

## 2. Material and methods

### 2.1. Transcriptome datasets and query sets

We retrieved complete transcriptome datasets for 126 crustacean species available at the time of manuscript preparation from the European Nucleotide Archive (https://www.ebi.ac.uk/ena). Six non-crustacean arthropod proteomes were retrieved from Uniprot (http://www.uniprot.org/). A complete list of accessions used in this study is provided in the electronic supplementary material, table S1. We retrieved a list of query sequences used in subsequent homology searches from Uniprot and GenBank.

### 2.2. Identification of bHLH orthologues

Based on a previously published workflow [36], we used multiple Basic Local Alignment Search Tool (BLAST)-based approaches such as BLASTp and tBLASTn with varying Blocks Substitution matrices to identify bHLH orthologues. The BLAST results were filtered by e-value of <10^−6^, best reciprocal BLAST hits against the GenBank non-redundant (nr) database and redundant contigs having at least 95% identity were collapsed using CD-HIT (https://github.com/weizhongli/cdhit). We then used HMMER employing hidden Markov models (HMM) profiles [37] to scan for the presence of bHLH Pfam domains [38] on the best reciprocal nr BLAST hits to compile a final non-redundant set of crustacean and arthropod bHLH orthologues. Pfam annotations and associated e-values are provided in electronic supplementary material, table S2.

### 2.3. Multiple sequence alignment and phylogenetic tree construction

Multiple sequence alignments of bHLH protein sequences were performed using MAFFT [39]. Phylogenetic trees were built from the MAFFT alignment using RAxML WAG + G model to generate best-scoring maximum-likelihood trees [40]. Bayesian inference trees were constructed using MrBayes [41]. Geneious was used to generate graphical representations of Newick trees [42].

## 3. Results and discussion

### 3.1. Annotation of putative bHLH genes in crustaceans

**Figure 1.**
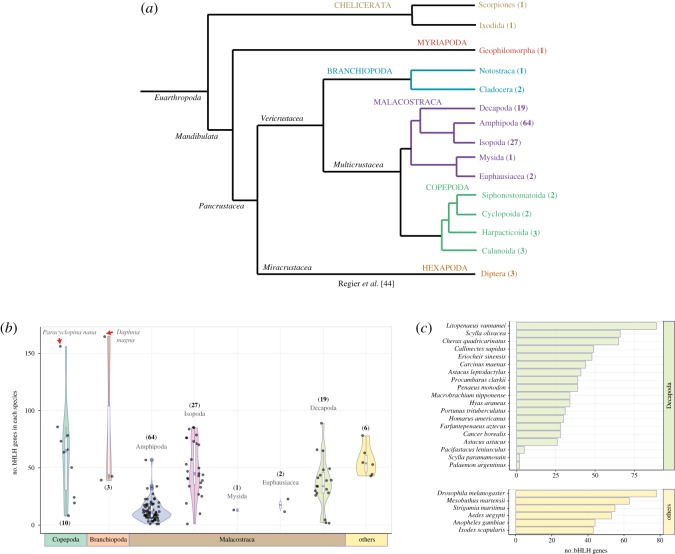
The bHLH superfamily in Crustacea and representative arthropod species. (*a*) Phylogenetic relationship of Arthropoda and Pancrustacea. The number of species within each taxon is denoted in parentheses. (*b*) Number of bHLH orthologues identified in each species is depicted as boxplots, indicating the median and quartiles. Violin plots underlying the boxplots illustrate sample distribution across different crustacean taxa and kernel probability density (width of the shaded areas represent the proportion of data located in these areas). The bHLH orthologues from six non-crustacean species within Arthropoda (others) are also shown. The number of species for each taxon is denoted in parentheses. Number of bHLH genes in *Paracyclopina nana* and *Daphnia magna* are marked with red arrows. (*c*) Bar charts illustrating the number of bHLHs in decapods and six non-crustacean arthropods (others).

Building on our previous analysis of bHLHs in the crustacean *Parhyale hawaiensis* [43], we have identified additional bHLHs from six non-crustacean arthropods: Insecta (three species), Arachnida (two species) and Chilopoda (one species) and 125 additional crustacean species representing three classes: Malacostraca (Amphipoda: 64 species, Decapoda: 19 species, Isopoda: 27 species, Euphausiacea: two species and Mysida: one species), Branchiopoda (three species) and Copepoda (10 species) (figure 1*a*; electronic supplementary material, tables S1 and S2 and file S1). From the complete genome sequences of model arthropod species, we annotated a total of 336 putative bHLHs; *D. melanogaster* (78 genes), *Aedes aegypti* (53 genes), *Anopheles gambiae* (44 genes), *Ixodes scapularis* (43 genes), *Mesobuthus martensii* (63 genes) and *Strigamia maritima* (55 genes) (figure 1). We have also identified 3777 bHLH genes from 126 crustacean species (including *P. hawaiensis*), hence providing a significant coverage of major Crustacea taxa (figure 1; electronic supplementary material, table S2 and file S1).

### 3.2. Assignment of bHLHs into higher-order groups/families

**Figure 2.**
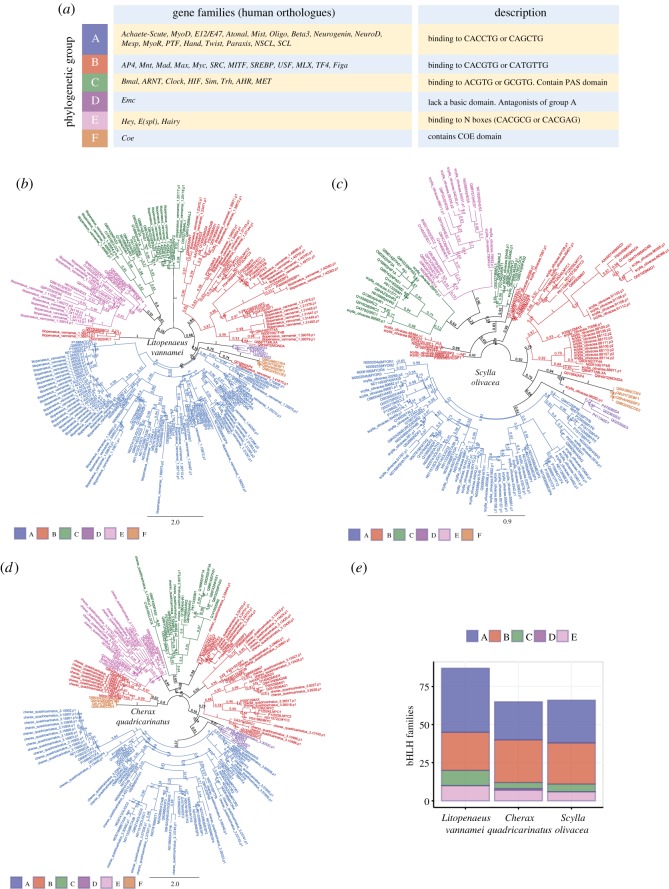
Classification of decapod bHLH proteins into higher-order groups. (*a*) The bHLH proteins can be further classified into six main groups (A–F) based on conservation of residues or the presence of additional domains [2,21,26]. Phylogenetic analyses of bHLHs from decapods (*b*) *Litopenaeus vannamei*, (*c*) *Scylla olivacea* and (*d*) *Cherax quadricarinatus.* Bootstrap support values (*n *= 1000) are denoted as branch labels. (*e*) Number of decapod bHLHs classified into groups A, B, C, D and E.

Considering the large number of putative bHLHs identified, independent analyses on genes from individual species are required. We therefore selected three decapod species for further analyses and assignment of bHLHs into higher-order groups. These species represent three dominant families of economically important food crops: Pacific whiteleg shrimp *Litopenaeus vannamei* (Penaeidae, 87 genes), freshwater crayfish *Cherax quadricarinatus* (Parastacidae, 65 genes) and mud crab *Scylla olivacea* (Portunidae, 66 genes). Maximum-likelihood trees were generated from multiple sequence alignments with bHLHs from *Homo sapiens* to define orthologous groups [21]. Overall, we observed that decapod bHLHs could be confidently assigned to previously described higher-order groups (A, B, C and E) (figure 2; electronic supplementary material, figure S1). Group A (proteins that bind CACCTG or CAGCTG E boxes) and group E (proteins that bind CACGCG or CACGAG N boxes) bHLHs are monophyletic in all three decapod species (figure 2*a–d*; electronic supplementary material, figure S1); a reappraisal of previous observations on bHLHs from human [21] and sponge [3]. Group B (proteins that bind CACGTG or CATGTTG E boxes) and group C (proteins that contain PAS domains) bHLHs are probably paraphyletic as they constitute members from other groups (figure 2*b*–*d*) [21]. We observed no evidence of group F bHLHs (figure 2*a*) and only one instance of a group D member from *C. quadricarinatus* (figure 2*d*,*e*). This could suggest that group D and F bHLH members are present but not represented in the transcriptome datasets, that members we have found have evolved convergently and/or a complex phenomenon of loss among certain crustacean taxa.

### 3.3. Evidence for lineage-specific duplications of bHLHs in Branchiopoda and Copepoda

Our analyses of the full bHLH gene complement from the genomes of seven species have revealed that arthropods/crustacean, on average, possess 56 bHLH genes: *Parhyale hawaiensis* (57 genes), *Ixodes scapularis* (43 genes), *Anopheles gambiae* (44 genes), *Aedes aegypti* (53 genes), *Strigamia maritima* (55 genes), *Mesobuthus martensii* (63 genes) and *Drosophila melanogaster* (78 genes); a finding consistent with another study [3]. Interestingly, we observed that two basal crustacean species, *Daphnia magna* (Branchiopoda) and *Paracyclopina nana* (Copepoda) have a significantly higher number of bHLH genes, 165 and 156 genes, respectively, than that of other crustacean and arthropod species (figure 1*b*; electronic supplementary material, table S2). This suggests that the expansion of the bHLH superfamily in these two species may have arisen through lineage-specific duplications. Phylogenetic analyses revealed that *P. nana* and *D. magna* bHLH genes could be assigned to four higher-order groups (A, B, C and E) with group E bHLHs exhibiting monophyly in both species (electronic supplementary material, figure S2 and S3). Others have reported that *Daphnia* has an unusually large collection of tandemly duplicated genes underpinning its intriguing phenotypic plasticity and remarkable ability to adapt to major ecological challenges [45]. The expansion of bHLHs as an adaptation mechanism to adverse environmental conditions can similarly be explained in copepods. These marine planktonic species serve as models for ecotoxicology and environmental genomics due to their high tolerance to a wide range of salinity and temperature [46–49]. As many bHLH members function as sensors of the environment, expansion through gene duplication would serve as an important evolutionary force for functional divergence to genetically adapt and cope with environmental stressors [50].

### 3.4. Monophyly of β-class bHLH-PAS proteins in decapods and basal crustaceans

**Figure 3.**
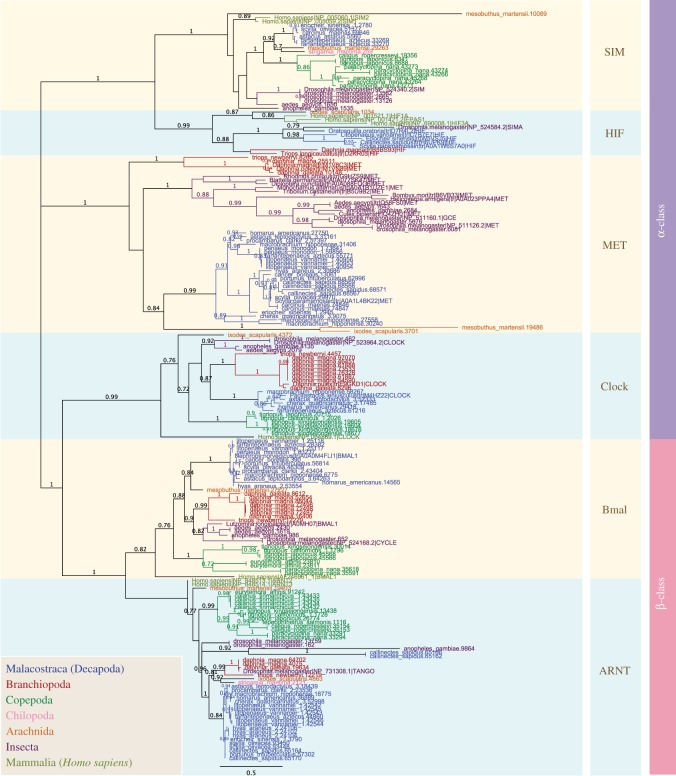
Phylogeny of the bHLH-PAS family in decapod, basal crustaceans and non-crustacean arthropods. The tree was constructed using the maximum-likelihood method from an amino acid multiple sequence alignment. The node labels of each taxon are marked with distinctive colours denoted in the figure inset. Bootstrap support values (*n *= 1000) above 0.7 are denoted as branch labels. The tree illustrates putative α-class and β-class members.

The period (PER)-ARNT-SIM (PAS) domain is found in proteins involved in recognizing and transducing environmental signals into appropriate cellular responses pertaining to stress signalling, development and circadian regulation. Many well-known PAS proteins have been shown to also possess a bHLH domain where they are collectively termed as bHLH-PAS [11,28]. The bHLH-PAS family is further classified into two subgroups: α-class and β-class [11,51]. The α-class proteins act as archetypal sensors of tissue-specific or environmental signals, e.g. single-minded (SIM-development), HIF (oxygen sensing), neuronal PAS domain proteins (NPAS-development) and circadian locomotor output cycles protein kaput (CLOCK-circadian timekeeping) and MET (juvenile hormone signalling) [11,51–53]. Unable to dimerise among themselves, α-class members require β-class proteins, aryl hydrocarbon receptor nuclear translocators (ARNT), as their broad-spectrum binding partners [11,51]. We perform phylogenetic analysis on bHLH-PAS members identified from decapods and basal crustaceans (branchiopods and copepods; figure 3; electronic supplementary material, figures S4 and S5). We have also included bHLH-PAS genes identified from other arthropod species (insects, tick, scorpion and centipede) and previously annotated proteins from Uniprot (figure 3; electronic supplementary material, figures S4 and S5) [21]. Consistent with other reports, we demonstrate that crustacean bHLH-PAS orthologues form two distinct phylogenetic groups, *α* and *β* (figure 3; electronic supplementary material, figures S4 and S5). The β-class ARNT proteins (including the BMAL family) form a well-supported monophyletic group, whereas α-class proteins are polyphyletic: HIF and SIM members form a single cluster while MET and CLOCK form separate monophyletic groups (figure 3; electronic supplementary material, figures S4 and S5). Our observation is consistent with previous reports suggesting that bHLH-PAS paraphyly could be explained through modular evolution [54]. Modular evolution by domain shuffling is commonly seen in bHLH proteins that also possess other highly conserved domains such as PAS. As evidenced by the lack of sequence similarity in flanking regions, it is likely that the association between PAS and bHLH domains occurred multiple times independently through domain duplication or insertion [19,54].

## 4. Conclusion

Although a majority of our analyses have focused on transcriptome datasets that are dependent on tissue-type and the differential expression of transcripts at the point of sample collection, the power of detection is supported by the fact that (i) the transcriptomes were sequenced to considerable depth (electronic supplementary material, table S1) and (ii) we do find consistent detection of bHLH gene families in whole-genome sequences (electronic supplementary material, table S2). We are unable to entirely rule out the possibility that some genes would remain undetected, hence potential caveats associated with transcriptome mining must be taken into consideration. In summary, we identified 3777 putative bHLH orthologues from 126 crustacean species representing major Crustacea taxa. We also annotated 336 bHLHs from six additional non-crustacean arthropods sampling across broad taxonomic range to include genomes of emerging model species (centipede and scorpion). We observed evidence for lineage-specific gene expansions in branchiopod and copepod suggesting a mechanism for genetic adaptation to adverse environmental factors commonly encountered by these species. Phylogenetic analyses on decapod bHLHs recapitulated the evolutionarily conserved higher-order orthologous groupings seen in other metazoans. Further analysis on group C bHLH-PAS members revealed that although β-class members are monophyletic, this is not true for the remaining α-class members.

## Supplementary Material

Supplementary table 1

## Supplementary Material

Supplementary table 2

## Supplementary Material

Supplementary file 1

## Data accessibility

Data supporting the conclusions of this study are provided as the electronic supplementary materials.
